# Genomic Epidemiology of 2015–2016 Zika Virus Outbreak in Cape Verde

**DOI:** 10.3201/eid2606.190928

**Published:** 2020-06

**Authors:** Oumar Faye, Maria de Lourdes Monteiro, Bram Vrancken, Matthieu Prot, Sebastian Lequime, Maryam Diarra, Oumar Ndiaye, Tomas Valdez, Sandra Tavarez, Jessica Ramos, Silvânia da Veiga Leal, Cecilio Pires, Antonio Moreira, Maria Filomena Tavares, Linete Fernandes, Jorge Noel Barreto, Maria do Céu Teixeira, Maria da Luz de Lima Mendonça, Carolina Cardoso da Silva Leite Gomes, Mariano Salazar Castellon, Laurence Ma, Frédéric Lemoine, Fabiana Gámbaro-Roglia, Déborah Delaune, Gamou Fall, Ibrahima Socé Fall, Mamadou Diop, Anavaj Sakuntabhai, Cheikh Loucoubar, Philippe Lemey, Edward C. Holmes, Ousmane Faye, Amadou Alpha Sall, Etienne Simon-Loriere

**Affiliations:** Institut Pasteur, Dakar, Senegal (Oumar Faye, M. Diarra, O. Ndiaye, G. Fall, M. Diop, C. Loucoubar, Ousmane Faye, A.A. Sall);; Ministerio da Saude, Praia, Cape Verde (M. de Lourdes Monteiro, T. Valdez, S. Tavarez, J. Ramos, S. da Veiga Leal, C. Pires, A. Moreira, M.F. Tavares, L. Fernandes, J.N. Barreto, M. do Céu Teixeira, M.L. de Lima Mendonça);; Katholieke Universiteit Leuven, Leuven, Belgium (B. Vrancken, S. Lequime, P. Lemey);; Institut Pasteur, Paris, France (M. Prot, L. Ma, F. Gámbaro-Roglia, D. Delaune, E. Simon-Loriere);; World Health Organization, Praia (C.C. da Silva Leite Gomes, M.S. Castellon, I.S. Fall);; CNRS USR 3756, Paris (F. Lemoine);; Université de Paris, Sorbonne Paris Cité, Paris (F. Gámbaro-Roglia);; Université Paris-Sud/Paris-Saclay, Orsay, France (D. Delaune);; Institut de Recherche Biomédicale des Armées, Brétigny-sur-Orge, France (D. Delaune); CNRS UMR 2000, Paris (A. Sakuntabhai);; The University of Sydney, Sydney, New South Wales, Australia (E.C. Holmes)

**Keywords:** Zika virus, microcephaly, Cabo Verde, Cape Verde, Brazil, West Africa, genomics, disease outbreaks, epidemiologic studies, phylogeography, epidemiology, outbreak, viruses, vector-borne infections, serology, Asian lineage

## Abstract

During 2015–2016, Cape Verde, an island nation off the coast of West Africa, experienced a Zika virus (ZIKV) outbreak involving 7,580 suspected Zika cases and 18 microcephaly cases. Analysis of the complete genomes of 3 ZIKV isolates from the outbreak indicated the strain was of the Asian (not African) lineage. The Cape Verde ZIKV sequences formed a distinct monophylogenetic group and possessed 1–2 (T659A, I756V) unique amino acid changes in the envelope protein. Phylogeographic and serologic evidence support earlier introduction of this lineage into Cape Verde, possibly from northeast Brazil, between June 2014 and August 2015, suggesting cryptic circulation of the virus before the initial wave of cases were detected in October 2015. These findings underscore the utility of genomic-scale epidemiology for outbreak investigations.

Zika virus (ZIKV), first discovered in Uganda in 1947 and sporadically found in Africa and Asia, was long believed to only cause mild disease in humans (*1*). ZIKV isolates are classified into 1 of 2 lineages, representing the African and Asian genotypes. ZIKVs of the African lineage have been isolated from many regions of Africa (*2*), mostly through entomologic investigations, and serologic evidence suggests that ZIKV infections in humans are frequent (*3*). However, until the 2000s, the virus had seldom been detected in humans. The Asian lineage has spread throughout the Pacific, causing outbreaks in humans in Yap, Federated States of Micronesia, in 2007 and in French Polynesia during 2013–2014, where an association with neurologic afflictions was first detected (*4*). Zika cases were first reported in Brazil in May 2015, and from there, the virus quickly spread to most of the Americas (*5*). The high number of cases led to the discovery of an association between congenital ZIKV infection and neonatal neurologic complications, particularly microcephaly (*6*,*7*).

In October 2015, an epidemic of rash, conjunctivitis, and arthralgia was noted by physicians in Praia, the capital of Cape Verde, an archipelago nation located in the Atlantic Ocean, west of the coast of Senegal. Blood samples sent to the regional reference laboratory of the Institut Pasteur de Dakar (Dakar, Senegal) confirmed the epidemic involved ZIKV infection. By the end of the outbreak in May 2016, a total of 7,580 suspected Zika cases and 18 microcephaly cases were reported in the 4 most densely populated southern islands of the Cape Verde archipelago (Brava, Fogo, Maio, and Santiago; Figure 1) (*8*). Overall, ≈50% of confirmed microcephaly cases were linked to reports of Zika-related symptoms in the mother during the first trimester of gestation (*8*).

**Figure 1:**
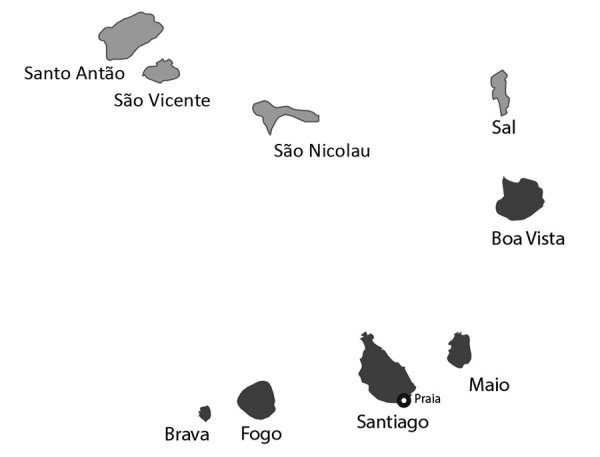
Locations of suspected Zika cases (dark gray shading), Cape Verde, 2015–2016. Only 2 cases on Boa Vista were confirmed, and those might have been imported.

## Experimental Procedures

### Sample Collection

In October 2015, Cape Verde reported a ZIKV outbreak and initiated a surveillance system to investigate the circulation of the virus in the country. All healthcare facilities were alerted to report suspected Zika cases according to the case definition of rash with or without fever and >1 of the following symptoms: conjunctivitis, headache, pruritus, arthralgia, myalgia, diarrhea, vomiting, adenopathy, or retro-orbital pain. In total, 1,226 sample sets (including blood and sometimes matching urine) of the original 7,580 sample sets from patients with suspected ZIKV infections (*9*) were sent to the Virology Laboratory at the Achadinha Health Center (Praia, Cape Verde) for ZIKV diagnosis. Only a fraction of the original sample set was sent because ZIKV testing was performed at the discretion of each healthcare facility’s medical practitioners. Staff also sent samples from patients not fitting the Zika case definition (i.e., patients with only rash or only fever) for Zika testing.

### Ethics

In this study, we used samples collected as part of approved ongoing surveillance conducted by the Institut Pasteur de Dakar (a World Health Organization Collaborating Centre for Arboviruses and Haemorrhagic Fever Reference and Research). All samples from humans were de-identified before we performed virus characterization and analyses; thus, no patient information can be reported.

### Molecular Tests

We tested acute serum samples (obtained <5 days after symptom onset; n = 387) and, when available, matched urine samples (n = 82) by quantitative reverse transcription PCR (qRT-PCR). We extracted RNA from serum or urine samples using the QIAamp Viral RNA Mini Kit (QIAGEN, https://www.qiagen.com) according to the manufacturer’s recommendations and performed a 1-step real-time PCR assay (*10*) on an ABI7500 instrument (Applied Biosystems, https://www.thermofisher.com) using the QuantiTect Probe RT-PCR Kit (QIAGEN).

### Serologic Tests

We tested serum samples (collected <10 days after symptom onset; n = 1,226) by ELISA for ZIKV IgM and IgG. For ZIKV antibody–positive samples, we tested for antibodies against other flaviviruses (yellow fever virus [YFV], dengue virus [DENV], and West Nile virus [WNV]) endemic in the West Africa region to rule out cross-reactions. 

For the IgM ELISA, we coated 96-well microtiter plates with a monoclonal IgM capture antibody (goat anti-human IgM; KPL, https://www.seracare.com) in carbonate bicarbonate buffer (pH 9.6) and incubated overnight at 4°C. After washing the plate 3 times with phosphate-buffered saline plus 0.05% tween, we added heat-inactivated (56°C, 30 min) patient serum samples and controls (all diluted 1:100 in phosphate-buffered saline plus 0.05% tween and 1% milk powder) in duplicate into plate wells and incubated at 37°C for 1 h. We washed wells 3 times; added ZIKV, DENV, WNV, or YFV antigens into plate wells; and incubated the plate for 1 h. After 3 washes, we added ZIKV-, DENV-, WNV-, or YFV-specific immune ascites from mice to each well and incubated for 1 h at 37°C. After 3 washes, we added peroxidase-labeled antibody specific to mouse IgG for 1 h at 37°C. Last, we added a tetramethylbenzidine substrate to the IgM conjugate complex and stopped the color reaction using a sulfuric acid solution. For the indirect IgG ELISA, we captured ZIKV antigen on 96-well plates coated with ZIKV-specific mouse hyperimmune ascitic fluid. We added patient serum samples (1:100) and then horseradish peroxidase–conjugated goat anti-human IgG. We considered serum samples positive if the optical density at 450 nm was >0.20 above the negative serum sample average and the ratio between the sample and the negative control was >2.

We analyzed samples positive for ZIKV IgM or IgG by ELISA for ZIKV-neutralizing antibodies using the plaque reduction neutralization test (PRNT) as described by De Madrid and Porterfield (*11*). In brief, we mixed 2-fold serial dilutions of serum samples (starting at 1:10) with equal volumes of medium containing 800 PFU/mL of the ZIKV reference strain MR766 and incubated for 1 h at 37°C. We then used serum–virus mixtures to infect Vero cell monolayers in 24-well plates. After 1 h at 37°C, we covered cells with DMEM containing 2% FBS and 0.4% carboxymethylcellulose and incubated for 4 days. We determined neutralizing antibody titers using an 80% cutoff value and classified samples as positive if their titers were ≥20 IU/mL.

### Untargeted Sequencing

We treated RNA samples first with Turbo DNase (Ambion, https://www.thermofisher.com) and then depleted host rRNA using the NEBNext rRNA Depletion Kit (New England Biolabs, https://www.neb.com). We used rRNA-depleted RNA samples for cDNA synthesis using random primers and Superscript IV (ThermoFisher, https://www.thermofisher.com) and used cDNA for library preparation (Nextera XT DNA Library Prep Kit; Illumina, https://www.illumina.com). We sequenced libraries on an Illumina NextSeq500.

### Amplicon-Based Sequencing

We sequenced amplicons from 3 samples following the protocol described by Quick et al. (*12*). We prepared sequencing libraries using the NEBNext Ultra II DNA Library Prep Kit for Illumina and barcoded with NEBNext Multiplex Oligos for Illumina (Dual Index Primers Set 1) (New England Biolabs for both). We sequenced prepared libraries on an Illumina MiSeq using MiSeq Reagent Kit v3 (600 cycles) at the Biomics platform of Institut Pasteur (Paris, France). We used Trimmomatic (http://www.usadellab.org) to remove adaptor and primer sequences (first 24 nt from 5′ end of reads, which is the maximum length of primers used for multiplexed PCRs). Then, we aligned reads to the complete genome sequence of a 2016 ZIKV isolate from the Dominican Republic (GenBank accession no. KU853012) using the CLC Genomics Suite (QIAGEN). We used SAMtools (http://samtools.sourceforge.net) to sort the aligned bam files and generate alignment statistics. We visually inspected ZIKV-aligned reads using Geneious version 9.1 (https://www.geneious.com) before generating consensus sequences and required a minimum of a 5× read-depth coverage to make a base call. We deposited all sequences we obtained in GenBank (accession nos. MK241415–7).

### Sequence Dataset Compilation

To determine the origin of ZIKV in Cape Verde, we created a comprehensive dataset of all publicly available ZIKV sequences from GenBank. We then removed from this dataset all laboratory, patented, African genotype, and pre-2000 Asian genotype isolates. We concatenated all genome fragments of the same isolate to retain 1 representative sequence per isolate. For sequences with incomplete information on sampling time and location in GenBank records, we attempted to retrieve this information from published studies and other online resources. For cases in which the origin of the imported case of ZIKV infection was known, we set the sampling location as the location of origin. Last, we removed sequences that were not linked to a publication or associated with a known sampling time or location. This data pruning resulted in a final dataset of 459 ZIKV sequences. We created a multiple sequence alignment of these data using MAFFT (*13*) and manually edited the alignment by using AliView (*14*).

### Phylogenetic Inference

We performed Bayesian phylogenetic and phylogeographic analyses using BEAST version 1.10.4 (*15*). We employed an uncorrelated relaxed-clock model (with rates drawn from an underlying log-normal distribution) (*16*) and a skygrid coalescent tree prior distribution (*17*) in combination with a codon-partitioned substitution model (SRD06) (*18*). We inferred migration history using a discrete phylogeographic model that incorporates a model-averaging procedure (i.e., the Bayesian stochastic search variable selection procedure) (*19*) to identify the subset of migration flows that adequately explain the diffusion process. We assessed convergence and mixing properties of the Markov chains with Tracer version 1.7 (*20*) and combined samples of several independent chains post burn-in. We generated maximum clade credibility trees summarizing the combined post burn in Markov chain Monte Carlo samples with the use of TreeAnnotator version 1.10.4 (http://beast.community/programs).

## Results and Discussion

**Figure 2:**
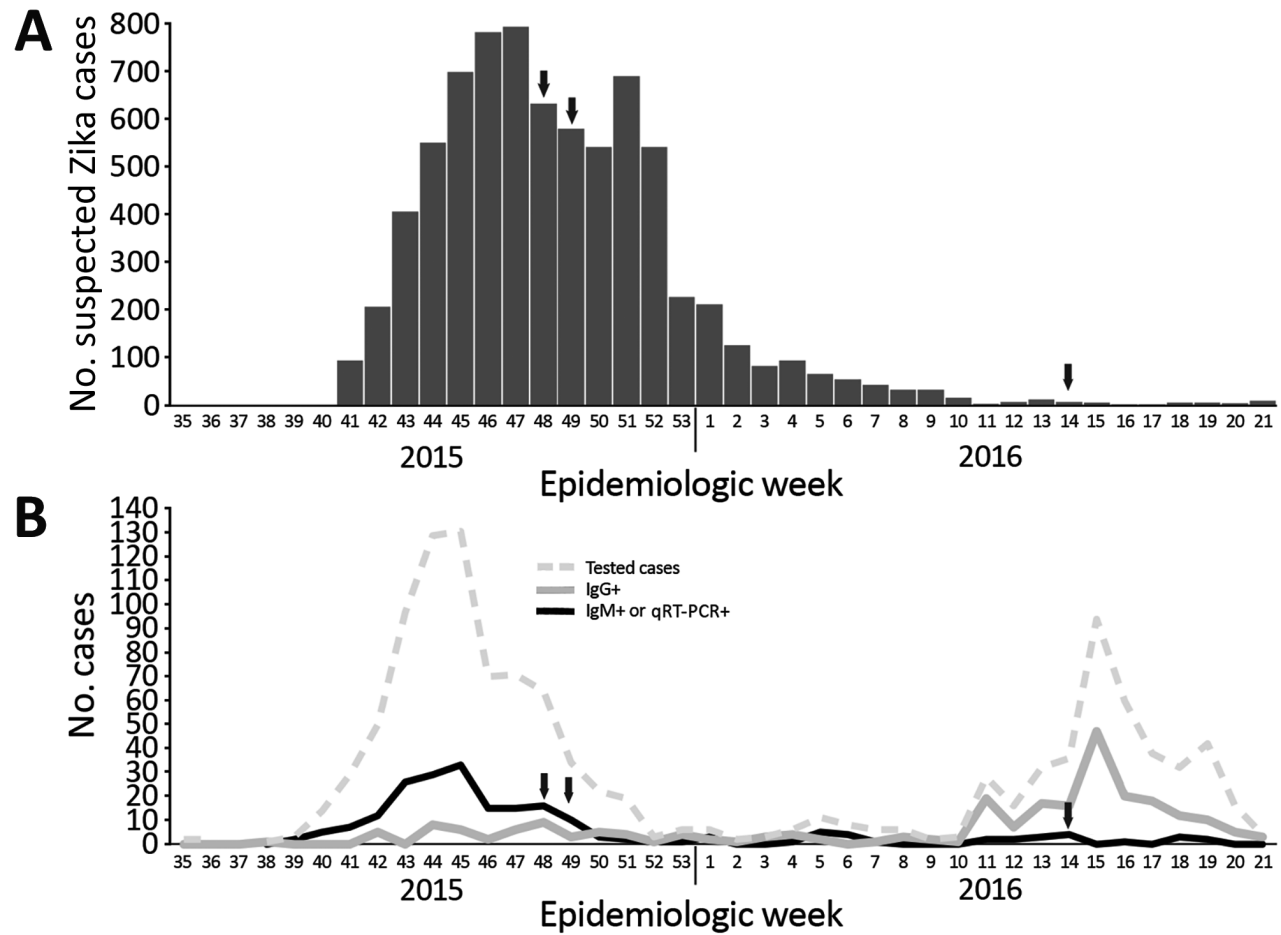
Suspected Zika cases, cases tested for Zika virus (ZIKV) infection, ZIKV antibody–positive cases, and ZIKV RNA–positive cases, Cape Verde, 2015–2016, by epidemiologic week. A) Cases of suspected ZIKV infection (n = 7,580) (*9*). B) Cases tested for ZIKV infection, ZIKV antibody–positive cases, and ZIKV RNA–positive cases. Only 1,226 of 7,580 cases of suspected ZIKV infection are included among those tested for ZIKV infection. In addition, some patients with fever only or rash only who did not fit the Zika case definition were also tested for ZIKV infection and included on this graph. ZIKV IgG–positive cases were negative by qRT-PCR and IgM ELISA and confirmed positive for ZIKV IgG by plaque reduction neutralization test. Arrows indicate the time of patient sampling for the 3 sequenced ZIKV isolates (GenBank accession nos. MK241415–7). qRT-PCR, quantitative reverse transcription PCR; –, negative; +, positive.

We report the findings of samples from 1,226 patients with suspected ZIKV infections (Table), as well as patients not fitting the Zika case definition. Samples were tested over the course of the Cape Verde outbreak. The practice of sending samples from cases not fulfilling the strict Zika case definition for ZIKV testing resulted in a surge of tests being performed during the latter part of the outbreak (Figure 2). The peak of acute infections (as detected by IgM ELISA or qRT-PCR) occurred 3 weeks before the peak of suspected cases. The presence of IgG-positive, IgM-negative, qRT-PCR–negative cases in April 2016 indicates that many infections were not detected during their acute phase and highlights the challenge of capturing the timing and extent of outbreaks when a large proportion of the infected population is asymptomatic. Indeed, 80% of ZIKV infections are asymptomatic (*21*). The early detection of samples positive for ZIKV IgG (confirmed by PRNT) and negative for ZIKV IgM and RNA (including samples with sampling dates before October 2015 that were also received for ZIKV testing though not fitting the Zika case definition) is compatible with the silent circulation of ZIKV before the major wave of reported cases. Although the high population densities of the southern islands (with >50% of the Cape Verde population living on Santiago Island) may explain why these islands experienced intense outbreaks, determining other factors that might have helped prevent further spread of the virus within the archipelago during the outbreak will be of value for future application.

**Table:** Confirmed Zika cases during 2015–2016 outbreak, Cape Verde*

Category	No. confirmed/no. tested
Confirmed recent ZIKV infections	226/1,226
qRT-PCR+/IgM–	18
qRT-PCR+/IgM+	6
qRT-PCR–/IgM+	202
Confirmed previous ZIKV infections: IgG+/PRNT+/qRT-PCR–/IgM–	311/1,226

*PRNT, plaque reduction neutralization test; qRT-PCR, quantitative reverse transcription PCR; ZIKV, Zika virus; –, negative; +, positive.

Before this outbreak on the Cape Verde archipelago, no Zika outbreaks or ZIKV-associated microcephaly cases had been reported in Africa, and only the Asian lineage of ZIKV had been found to be associated with neurologic and congenital afflictions. Because Cape Verde is situated in an area (near Senegal) where the African lineage of ZIKV has been regularly detected (*22*) and the Cape Verde outbreak involved cases of microcephaly, we needed to determine the origin and genotype of the ZIKV that caused this outbreak. We assessed virus origin and genotype through sequencing and phylogenetic analysis of ZIKVs sampled from patients in Cape Verde. Although our attempts at untargeted sequencing with rRNA-depleted samples were unsuccessful, we were able to Sanger sequence a small fragment of the envelope gene, which revealed that the virus was a part of the Asian lineage. We shared this information with the World Health Organization in May 2016.

**Figure 3:**
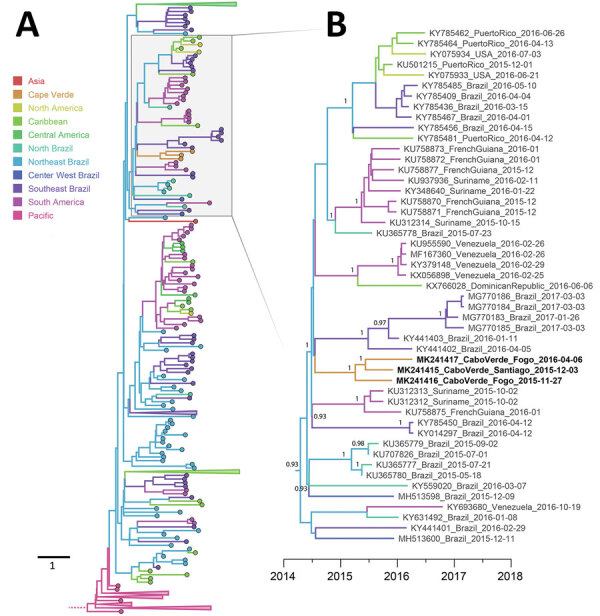
Maximum clade credibility phylogenetic tree demonstrating migration history of Zika virus (ZIKV) Asian lineage, 2014–2018. A) Phylogeny of 459 ZIKV isolates. The tree base was removed for ease of presentation. Tips of tree are colored according to their sampling location and branches according to their most probable geographic location. Note that sequences from the 2016 Angola outbreak (*23*) were published during the later stages of preparation of this manuscript and therefore were not included in this Bayesian analysis. Scale bar indicates years. See Appendix Figure 1 for fully annotated tree. B) Expansion of tree containing Cape Verde ZIKV sequences (bold). Clade posterior probabilities are shown at well-supported nodes (>0.9). GenBank accession number, country of origin, and sampling date are provided for each ZIKV sequence.

We subsequently used a published overlapping PCR amplicon scheme to amplify and sequence the ZIKV genomes of preserved samples (*12*). This procedure enabled us to construct libraries that we could sequence and resulted in us obtaining 3 complete ZIKV genomes. These 3 sequences were obtained from samples acquired in November 2015 (Fogo), December 2015 (Santiago), and April 2016 (Fogo). Bayesian phylogenetic analysis of publicly available ZIKV sequences indicated that all 3 Cape Verde ZIKV genomes were related to the Asian genotype and formed a single, separate, well-supported monophyletic group (posterior probability 1, Figure 3; Appendix Figure 1), confirming our initial inference that was based on a partial envelope gene fragment. Of note, all sequences of Cape Verde ZIKVs contained a unique nonsynonymous substitution in the envelope protein (I756V), and a second amino acid change (T659A) also in the envelope gene was present in 2 of the isolates (those acquired December 2015 and April 2016). Additional work is needed to determine whether these mutations arose by chance or if they facilitate adaptation to local conditions, such as growth in mosquito species of Africa.

Phylogeographic analyses indicated that the Cape Verde ZIKV clade shares a common ancestor with lineages circulating in Brazil (posterior probability 0.798) and is most likely from northeast Brazil (posterior probability 0.574). Both Brazil and Cape Verde are Portuguese-speaking countries, and direct flights between northeast Brazil (Fortaleza) and Cape Verde (Praia) occurred daily during the outbreak. Although our data suggest that the ZIKV responsible for the Cape Verde outbreak originated in northeast Brazil, a lack of genomic sampling from many ZIKV-affected regions in the Americas makes excluding transmission through these other locations impossible. The median estimated date for the most recent common ancestor of the Cape Verde ZIKV clade was June 2015 (95% highest posterior density March 2015–August 2015; Figure 3). The median estimated date of divergence of this clade from the sequences sampled outside Cape Verde was November 2014 (95% highest posterior density July 2014–March 2015). Hence, these results indicate that ZIKV was most likely introduced into Cape Verde between June 2014 and August 2015, clearly before the first wave of documented clinical cases. This estimated virus introduction date is also supported by the finding of persons with ZIKV IgG and neutralizing antibodies early during the outbreak (Figure 2).

This study has 2 main limitations. First, because only a fraction of suspected Zika case samples were tested, bias in the reported distribution of confirmed cases is possible. Second, only 3 ZIKV genome sequences were recovered from this outbreak, perhaps limiting the power of our phylodynamic and phylogeographic analyses.

In conclusion, our analysis indicates that the Asian lineage of ZIKV reached Cape Verde in 2015 and triggered a relatively large epidemic. Of note, the Cape Verde ZIKV clade is distinct from the ZIKV of the 2017 Angola outbreak (Appendix Figure 2), which also involved microcephaly cases (*23*,*24*). The introduction of ZIKV into Cape Verde from Brazil and silent circulation in Cape Verde was likely to have occurred before the wave of cases in October 2015, as revealed by both phylogenetic and serologic data. The time scale we report is consistent with those estimated for the initial movement of ZIKV from Brazil to neighboring countries in South America and islands of the Caribbean (Appendix Figure 1) (*10*,*25*,*26*).

Appendix Additional information about genomic epidemiology of 2015–2016 Zika virus outbreak in Cape Verde.
