# Supplementary material for: Genomic Epidemiology of 2015–2016 Zika Virus Outbreak in Cape Verde
Source: Emerg Infect Dis. 2020 Jun;26(6):1084–90. doi: 10.3201/eid2606.190928 (PMC7258482; doi:10.3201/eid2606.190928)
Supplement: Appendix — Additional information about genomic epidemiology of 2015–2016 Zika virus outbreak in Cape Verde. [file 19-0928-Techapp-s1.pdf]

Article DOI: https://doi.org/10.3201/eid2606.190928

Genomic Epidemiology of 2015–2016 Zika Virus Outbreak in Cape Verde

Appendix

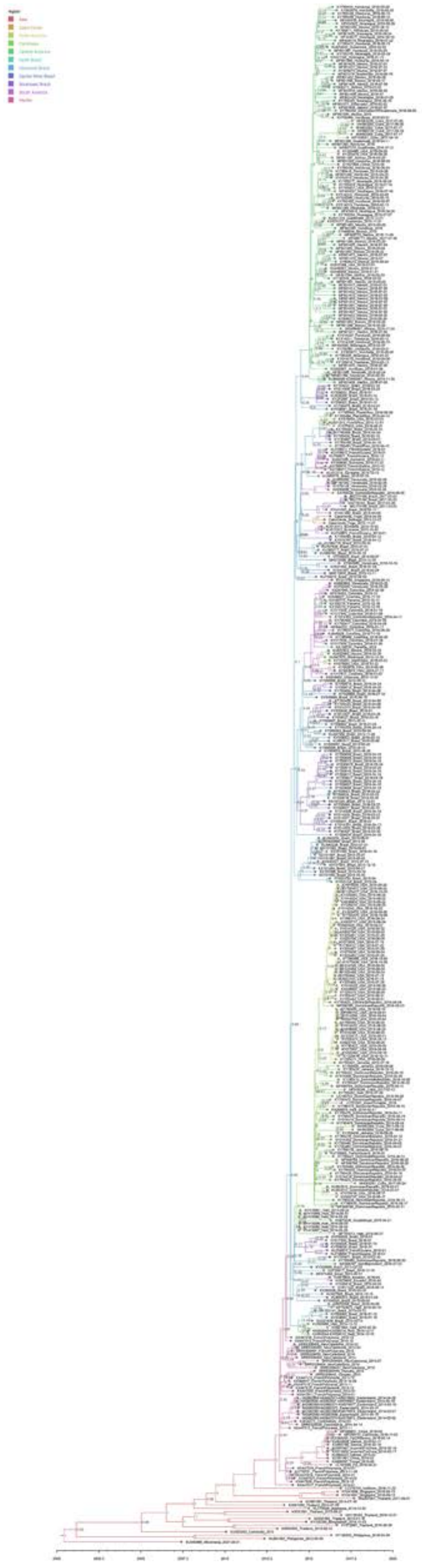

Appendix Figure 1. Maximum clade credibility phylogeny. The tips of the tree are colored according to their sampling location, and branches are colored according to their most probable geographic location: Asia (red); Cabo Verde (orange); North America (yellow); Caribbean (olive green); Central America (green); North Brazil (sea green); Northeast Brazil (light blue); Centre West Brazil (blue); Southeast Brazil (purple); South America (pink); Pacific (magenta). Posterior probabilities are shown at each node on the tree. Note that sequences from the 2016 Angola outbreak (*1*) were published during the later stages of this manuscript preparation and were therefore not included in the Bayesian analysis.

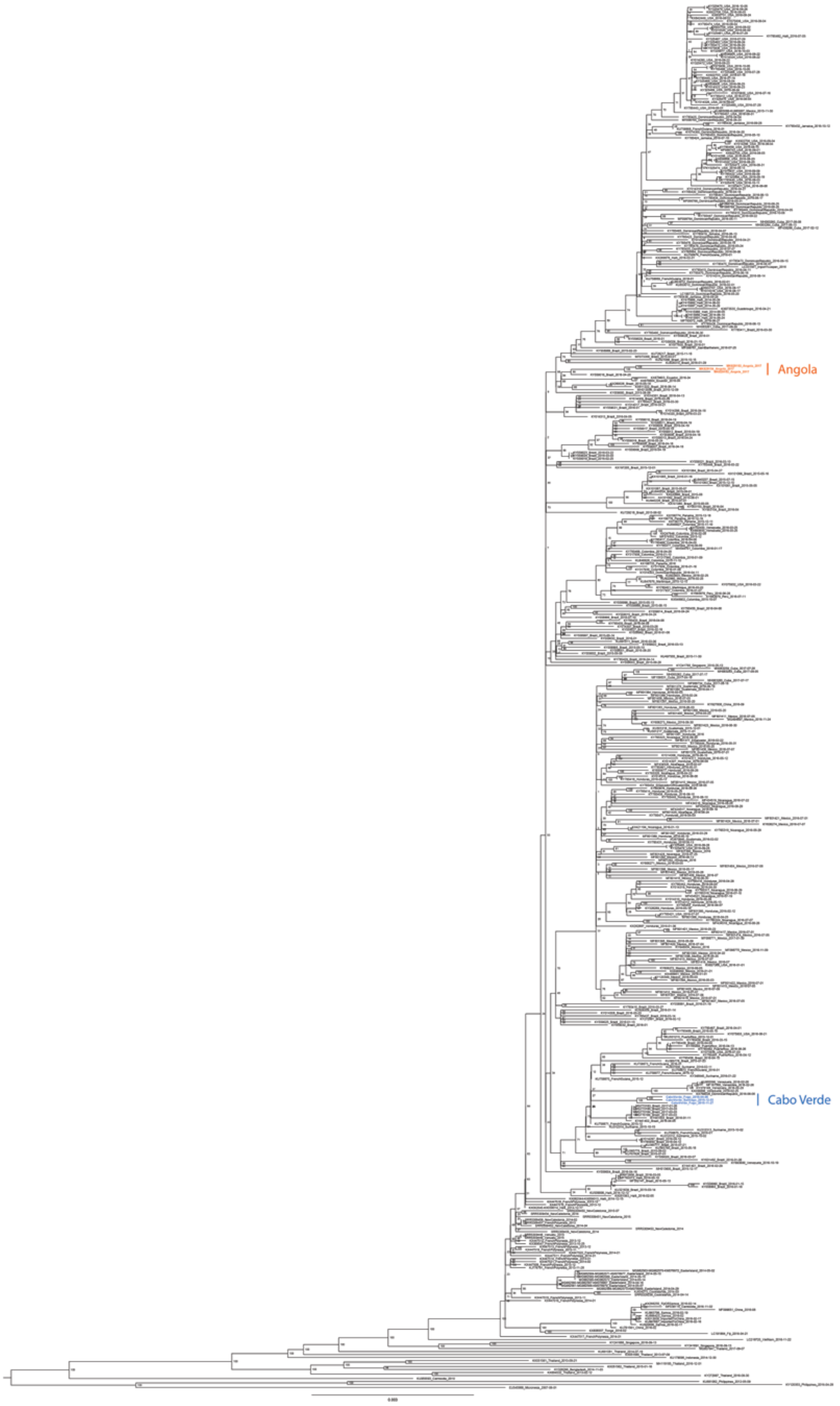

Appendix Figure 2. Maximum likelihood phylogeny of the Zika virus. The tree was estimated with IQ-Tree 1.6.7.2 using 459 complete and partial genomes (including three genomes from Angola). The Angola and Cabo Verdean clusters are colored in orange and blue, respectively. Bootstrap supports (ultrafast bootstrap approximation) are shown at each node on the tree.

Reference

1. Hill SC, Vasconcelos J, Neto Z, Jandondo D, Zé-Zé L, Aguiar RS, et al. Emergence of the Asian lineage of Zika virus in Angola: an outbreak investigation. Lancet Infect Dis. 2019;19:1138–47. PubMed http://dx.doi.org/10.1016/S1473-3099(19)30293-2
